# Higher congenital transmission rate of *Trypanosoma cruzi* associated with family history of congenital transmission

**DOI:** 10.1590/0037-8682-0560-2019

**Published:** 2020-04-27

**Authors:** Emmaría Danesi, Diana Lucrecia Fabbro, Elsa Leonor Segura, Sergio Sosa-Estani

**Affiliations:** 1 Centro Nacional de Diagnóstico e Investigación en Endemo-epidemias, Administración Nacional de Laboratorio e Institutos de Salud, Buenos Aires, Argentina.; 2 Universidad Nacional del Litoral, Centro de Investigaciones sobre Endemias Nacionales, Facultad de Bioquímica y Ciencias Biológicas, Santa Fe, Argentina.; 3 Instituto Nacional de Parasitología “Dr. Mario Fatala Chaben”, Administración Nacional de Laboratorio e Institutos de Salud, Buenos Aires, Argentina.; 4 Epidemiology and Public Health Research Center, CONICET, Buenos Aires, Argentina.

**Keywords:** Trypanosoma cruzi, Congenital Chagas disease, Vertical infection, Vertical transmission, Family clustering, Epidemiology

## Abstract

**INTRODUCTION:**

Congenital transmission (CT) of *Trypanosoma cruzi* has led to globalization of Chagas disease and its growing relevance as a public health problem. Although the occurrence of CT has been associated with several factors, its mechanisms are still unknown. This study aimed to analyze the geographical and familiar variables of mothers and their association with CT of Chagas disease in a population living in non-endemic areas of Argentina for the last decades.

**METHODS:**

We developed a retrospective cohort study in a sample of 2120 mother-child pairs who attended three reference centers in the cities of Buenos Aires, Santa Fe, and Salta between 2002 and 2015.

**RESULTS:**

The highest CT rates were observed in children born to Argentinean mothers (10.7%) and in children born to mothers from Buenos Aires (11.7%). Considering the areas of origin of the mothers, those from areas of null-low risk for vector-borne infection had higher CT rates than those from areas of medium-high risk (11.1% vs 8.2%). We also observed a significant intra-familiar “cluster effect,” with CT rates of 35.9% in children with an infected sibling, compared to 8.2% in children without infected siblings (RR=4.4 95% CI 2.3-8.4).

**CONCLUSIONS:**

The associations observed suggest a higher CT rate in children born to mothers who acquired the infection congenitally, with familiar antecedents, and from areas without the presence of vectors. These observations are considered new epidemiological evidence about Chagas disease in a contemporary urban population, which may contribute to the study of CT and may also be an interesting finding for healthcare professionals.

## INTRODUCTION

According to the estimations of the World Health Organization, infection by *Trypanosoma cruzi*, the etiological agent of Chagas disease (CD), affects over 6-8 million people worldwide, 5.5 million of whom live in Latin America^1^ ^-^ ^2^. Argentina is the country with the largest number of persons infected with CD, and annually, there are over 1500 congenitally infected newborns^1^.

Since vector-borne and transfusional transmissions have been reduced, congenital transmission (CT) has become the main route of generation of new cases of CD in several countries and, thus, is considered an important matter of public health. Moreover, because of CT and migrations, CD is now observed in urban areas and regions known to have no presence of the CD vectors in all continents^2^ ^-^ ^4^.

The mechanisms involved in CT are still unknown, and its occurrence cannot be predicted. Nevertheless, several studies have observed the interactions between the following different factors that may contribute to the mechanisms of CT: (i) parasitological aspects, (ii) the maternal immune system, (iii) placental physiology, and (iv) the fetal immune system, determined genetically and by environmental interactions^5^ ^-^ ^6^.

Different regions of Latin America and the world present different rates of CT, a fact associated with the prevalence of diverse *T. cruzi* discrete typing units (DTUs), named TcI to TcVI^7^. A recent study, for example, has found that both Argentina and Mexico present similar CT rates (approximately 6.5%) and that mothers of both countries are predominantly infected by non-TcI *T. cruzi* ^8^, presumably TcV. At the same time, in Brazil, where TcII and TcVI are the predominant DTUs in the domestic cycle, the rate of CT is approximately 1%^9^. Studies conducted in Chile, Bolivia, and Argentina have shown that parasites in mothers and their children are of the same DTU, with similar prevalent DTU in the general population^10^ ^-^ ^12^. Burgos et al.^12^ observed no differences between the genotypes of mothers with infected children and those of mothers with non-infected children. In an experimental study with placental explants and two strains of DTUs, TcII and TcVI, Medina et al.^13^ found that the latter had more infective capacity and pathogenicity than the former.

Regarding the parasitemia levels, several studies have shown a higher CT rate in women with higher levels of parasitemia than women with lower levels of parasitemia^15^ ^-^ ^18^, postulating this as the principal risk factor. Additionally, various studies have shown that trypanocidal treatment before pregnancy drastically reduces the occurrence of CT, which may be explained by a reduction in the parasitic load in the peripheral blood^19^ ^-^ ^23^. Additionally, according to Howard et al.’s^23^ meta-analysis, the CT rate in non-endemic countries is approximately 2%, whereas in endemic countries, it is between 4% and 5%. It has been suggested that these higher CT rates in endemic countries might be due to higher parasitic loads as a possible result of multiple maternal reinfections^24^ ^-^ ^25^. However, a study conducted in the City of Santa Cruz de la Sierra in Bolivia found that women who had never been exposed to the vector had a CT rate of 16.1% in comparison to 9.0% in women who had lived in houses with the presence of vectors^26^. The study also found that the former had higher parasitic loads than the latter. Similar findings were obtained in a study with women residing in Santa Fe, Argentina^27^. The authors of these studies hypothesize that reinfections in rural areas could activate the immune response with the consequent control of parasitemia.

Other studies have shown the importance of the immune system in the control of parasitemia and how higher CT rates are associated with deficiencies in gamma interferon and certain interleukins involved in cellular immunity^17^ ^,^ ^28^.

Additionally, some researchers have observed the occurrence of a “cluster effect” of a higher CT rate through generations of the same family and from a mother to several of her children^26^ ^-^ ^30^. However, these observations were limited considering the small sample size in this study.

In the present study, we focused on some maternal characteristics, such as their geographical origin and family history, to analyze their association with CT of CD in a contemporary population in an urban area of Argentina.

## METHODS

Our study was conducted on a retrospective cohort on a secondary database of 2120 children and their mothers chronically infected with *T. cruzi* who were living in urban areas of Argentina, in both non-endemic and endemic regions under control of vector transmission. The database was constructed from records of three reference centers: the Instituto Nacional de Parasitología (INP) (n=1854), the Centro de Diagnóstico e Investigación en Endemoepidemias (CeNDIE) (n=41) in the City of Buenos Aires (CABA) (most cases of CeNDIE were from studies performed in Salta Province), and the Centro de Investigaciones sobre Endemias Nacionales (CIEN) (n=225) in the City of Santa Fe. Records were obtained from mothers and their children who attended to the mentioned centers for controls of congenital CD or who participated in particular projects in the past. The information in the records was obtained by physicians or trained personnel. Mothers who received trypanocidal treatment prior to pregnancy were excluded. All available patients who had completed the diagnostic follow-up (approximately 70%) were included in the database. A case of congenital infection was considered when children with no exposure to vectors had positive parasitological tests or two reactive serological tests after 10 months of age, which were performed at the centers mentioned. We analyzed the association between the infection in the child and the following maternal variables: age at delivery, country and province of birth, risk of vector-borne transmission (VR) in the place of birth, place of current residence, infection of the mother (i.e., infection of the maternal grandmother of the child), and other children infected (brother or sister of the child). The VR variable was generated considering the year of birth of the mother and the categorization of the place of birth mentioned in the literature and programs of vectorial control^32^ ^-^ ^34^. The category of null VR included non-endemic areas, the category of low VR included endemic areas with certification of interruption of vector-borne transmission after 1970, and the categories of high and medium VR included areas of high or medium historical endemicity, without certification of interruption of vector-borne transmission after 1970.

Regarding the continuous variables, we calculated the central tendency parameters (interquartile ranges, median, mean) for infected and non-infected groups and performed Student’s t-test to analyze for significant differences. Regarding the categorical variables, we calculated the frequencies according to groups of infection and performed chi-squared or Fisher’s exact tests as necessary. Relative risks (RRs) of congenital infection with their confidence intervals (CIs) were calculated for variables that showed significant differences between groups. The significance level was set at 0.05. Analyses were performed using the Stata 11 software.

Ethical considerations. The databases used for this study were secondary data without any personal identification of contact information, and each case had an identification code previously assigned. The protocol was approved by the scientific committee of the institution in charged to conduct the study.

## RESULTS

**FIGURE 1:**
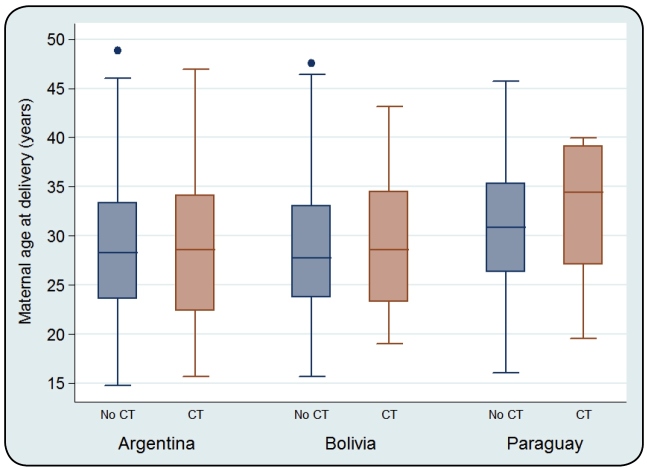
Maternal age at delivery, in groups according to maternal country of origin and congenital infection of children. **Source:** Own elaboration. **Notes:** No CT: Pair without congenital transmission; **CT:** Pair with congenital transmission.

Out of the 2120 children included in the study, 90% were born between 2002 and 2015 and lived mainly in the CABA (19.5%) and Province of Buenos Aires (PBA, 72.8%), the main metropolitan areas of Argentina. Congenital infection with *T. cruzi* was confirmed in 9% (192/2120) of children. Maternal age at delivery was 28.9±6.6 years in average, with a normal distribution and a range of 14.8-48.8 years. There was no significant difference in the age of mothers with infected and non-infected children (Student’s t-test, p=0.7389), and the distribution of ages in groups of nationalities was also similar (Figure 1). Regarding the country of birth, 60.2% of the mothers were born in Argentina, 29.1% in Bolivia, 10.3% in Paraguay, and 0.6% in other countries. The CT rate of children born to Argentinean mothers was higher (10.7%) than that of children born to mothers from other countries (chi-squared test, p=0.003, Table 1). In children born to Argentinean mothers, the risk of being infected was 50% higher than in children born to Bolivian mothers (RR=1.5; 95% CI, 1.1-2.1) and approximately twofold higher than that in children born to Paraguayan mothers (RR=2.1; 95% CI, 1.2-3.9).

**TABLE 1:** Distribution of mother-child pairs according to congenital infection by *Trypanosoma cruzi* and variables on geographical origin and family history.

	Total	No CT	CT	P
	N (%)	N (%)	N (%)	
Total population (sample)	2120 (100.0)	1.928 (90.9)	192 (9.1)	
Country of maternal origin (n=2111)				
Argentina	1275	1.138 (89.3)	137 (10.7)	0.003
Bolivia	617	573 (92.9)	44 (7.1)	
Paraguay	219	208 (95.0)	11 (5.0)	
Argentinian province of maternal origin (n=869)				
Buenos Aires	375	331 (88.3)	44 (11.7)	0.063
CABA	183	163 (89.1)	20 (10.9)	
Chaco	186	171 (91.9)	15 (8.1)	
Santiago del Estero	125	120 (96.0)	5 (4.0)	
Vectorial risk of area of maternal origin (n=2076)				
High	1001 (405)	926 (92.5)	75 (7.5)	0.073
Medium	152	364 (89.9)	41 (10.1)	
Low	521	135 (88.8)	17 (11.2)	
Null (non-endemic area)		463 (88.9)	58 (11.1)	
Vectorial risk of area of maternal origin (n=2076)				
Medium-high	1406	1.290 (91.8)	116 (8.2)	0.033
Low-null	673	598 (88.9)	75 (11.1)	
Maternal grandmother infected with *T. cruzi* (n=927)				
Yes	457	394 (86.2)	63 (13.9)	0.001
No	470	436 (92.8)	63 (7.2)	
Any sibling infected congenitally (n=198)				
Yes	64	41 (64.1)	23 (35.9)	<0.000
No	134	123 (91.8)	11 (8.2)	

**Source:** Own elaboration. **Notes:** No CT: Pair without congenital transmission; CT: Pair with congenital transmission.

Argentinean women were born in 20 of the 24 provinces, mainly in PBA (29.4%), Chaco (14.6%), CABA (14.4%), and Santiago del Estero (9.8%) (Annex Table 1). The proportion of infected children born to mothers from Buenos Aires (from both PBA and CABA) was higher than 10%, followed by 8.1% of children born to mothers from Chaco and 4.0% of children born to mothers from Santiago del Estero (Table 1). The difference in the CT rate between these groups was not statistically significant (chi-squared test, p=0.063). Considering the stratification by VR, we observed a higher frequency of cases of CT in the stratum of low-null VR, with a rate of 11.1%, which was statistically different from the stratum of medium-high VR (chi-squared test, p=0.033, Table 1).

**ANNEX TABLE 1:** Geographical origin of mothers.

	Total	No CT	CT
	N (%)	N (%)	N (%)
Total population (sample)	2120 (100.0)	1928 (100.0)	192 (100.0)
Country of maternal origin (n=2119)			
Argentina	1275 (60.2)	1138 (59.1)	137 (71.4)
Bolivia	617 (29.1)	573 (29.7)	44 (22.9)
Brazil	3 (0.1)	3 (0.2)	0 (0.0)
Chile	1 (0.1)	1 (0.1)	0 (0.0)
Paraguay	219 (10.3)	208 (10.8)	11 (5.7)
Perú	1 (0.1)	1 (0.0)	0 (0.0)
Uruguay	2 (0.1)	2 (0.1)	0 (0.0)
Otros	1 (0.0)	1 (0.0)	0 (0.0)
Argentinean province of maternal origin (n=1275)			
Buenos Aires	375 (29.4)	331 (29.1)	44 (32.1)
CABA	183 (14.3)	163 (14.3)	20 (14.6)
Catamarca	2 (0.2)	2 (0.2)	0 (0.0)
Chaco	186 (14.6)	171 (15.0)	15 (10.9)
Corrientes	29 (2.3)	27 (2.4)	2 (1.5)
Córdoba	6 (0.5)	6 (0.5)	0 (0.0)
Entre Ríos	26 (2.0)	19 (1.7)	7 (5.1)
Formosa	34 (2.7)	30 (2.6)	4 (2.9)
Jujuy	48 (3.8)	42 (3.7)	6 (4.4)
La Pampa	2 (0.6)	2 (0.2)	0 (0.0)
La Rioja	1 (0.1)	1 (0.1)	0 (0.0)
Mendoza	13 (1.0)	11 (1.0)	2 (1.5)
Misiones	31 (2.4)	27 (2.4)	4 (2.9)
Neuquén	3 (0.2)	3 (0.3)	0 (0.0)
Rio Negro	2 (0.2)	2 (0.2)	0 (0.0)
Salta	58 (4.5)	51 (4.5)	7 (5.1)
San Juan	6 (0.5)	5 (0.4)	1 (0.7)
San Luis	3 (0.2)	3 (0.3)	0 (0.0)
Santa Fe	119 (9.3)	99 (8.7)	20 (14.6)
Santiago del Estero	125 (9.8)	120 (10.5)	5 (3.7)
Tucumán	23 (1.8)	23 (2.0)	0 (0.0)

**Source:** Own elaboration.

Regarding familiar antecedents, 56% of the mothers (1179/2106) did not know the state of infection of their own mother, and of those that knew it, 49.3% answered affirmatively. The CT rate in the children with an infected grandmother was significantly higher than that in those whose grandmother was not infected (13.8% vs 7.23%, p=0.001). In a subgroup of mother-child pairs (n=198), information on infection of siblings was available. In the group of infected children (n=34), 67.6% had one or more infected siblings, while in the group of non-infected children (n=164), this proportion was 25.0%. The CT rate in children with a precedent of an infected sibling (35.9%) was significantly different from that in those without an infected sibling (8.2%) (chi-squared test, p=0.000). This implies that children with an infected sibling have four times higher risk of infection than those without an infected sibling (RR=4.4; 95% CI, 2.3-8.4).

## DISCUSSION

This study focused on a contemporary population of Buenos Aires, the main metropolitan area of Argentina, which is an urban vector-free area, where CD is not perceived as existing or as a relevant issue by healthcare professionals and by different levels of policy-makers. We observed that women with CD were born in almost all provinces of Argentina, not only in those endemic for the vector or rural areas but also in urban and non-endemic areas. Almost half of the Argentinean women studied were born in Buenos Aires (CABA and PBA); hence, we can assume that they had acquired the infection by CT and did not have adequate access to the diagnosis and treatment of CD. This may also be associated to the luck of awareness of the mother’s state of infection mentioned in more than half of the women. This should be considered by healthcare teams to eliminate the belief that people with CD are only those migrating from rural or historically endemic localities, particularly in Argentina to reinforce the need to accomplish the guidelines of universal control for CD in pregnant women and children born to infected mothers.

The CT rate observed in Argentinean women was higher than that in Bolivian and Paraguayan women, a result consistent with results of other studies^16^ ^,^ ^23^ ^,^ ^35^. In cases of differences in CT rates, these may be explained by the diversity in the age of the newborn and diagnostic techniques used. The results of this study contribute to the hypothesis that differential CT rates are associated with the prevalence of different parasite lineages.

Several researchers have mentioned that the principal risk factor for CT is the high parasitemia in pregnant women^14^ ^-^ ^17^. We may suppose that those women are from endemic areas, with higher probability of reinfection by vector exposure, and younger women with more recent infections. When we analyzed the mother’s age at delivery in relation to CT, we found no significant differences, in contrast to that previously reported by other authors^17^ ^,^ ^31^. When we considered the variables of maternal geographical origin, we surprisingly found that children born to women from non-endemic areas had the highest CT rates. This was observed mainly in children born to Argentinean women and in children born to women from Buenos Aires (CABA and PBA). Furthermore, when considering the variable of VR, the group with the highest CT rate was that of low-null VR. As mentioned above, considering the country of origin of the mother, we also found higher CT rate in children born to Argentinean women, half of whom were from non-endemic areas. Although Paraguay and Bolivia have substantially improved vector control, this process is recent and hence, women included in this study were considered to be from medium-high VR areas.

These observations may lead to the hypothesis that at ecological level, women originally from endemic areas (resident in non-endemic areas) would not have higher parasitemia and therefore no higher risk for CT. This hypothesis is consistent to what was observed by Rendell et al. in Bolivia^26^, where women who had been exposed to the vector showed lower parasitic load in the peripheral blood and lower CT rate than women who had not been exposed. Another possibility is that exposure to a higher load of parasites, reinfection, or the vector-mediated infection would have led to a process of adaptation, equilibrium, and protection to the placental passage of the parasite. Since this study did not include data on parasitological exams, further studies should confirm this epidemiological observation and this hypothesis. However, excluding the assumption of a higher parasitemia caused by vector-borne reinfection, it is striking that women born in areas of medium-high VR showed a lower CT rate and not, in any case, the same rate as women from areas of low-null VR. Women born and raised in non-endemic areas would have the particularity of having acquired the infection exclusively by congenital route (or exceptionally by contaminated transfusion), whereas those from endemic areas would have acquired the infection by both the congenital and vectorial routes of transmission. The studies of Sanchez-Negrette et al. conducted in Salta^29^ and Suasnábar et al. in Santa Fe^27^, both in Argentina, also presented similar observations, showing that women from rural areas of high endemicity had lower CT rates than women from urban areas not exposed to the vector. Sanchez-Negrette et al. proposed the occurrence of a “cluster effect,” which suggests that women from areas of lower VR may be infected by strains with a greater “capacity for congenital transmission,” thus giving place to transmission from one generation to another or to several children. This would be a selection process leading to the prevalence of “more transmissible” strains. This selection process would be more evident in urban areas where no vector-borne infections take place, and thus, is avoided the presence of other strains that at population level may result in lower CT rates. Medina et al.^13^ conducted an *in vitro* study in which they used a strain isolated from a patient with congenital infection and found that it had greater virulence for CT compared with another strain of a different genotype. This is the first relevant evidence of the hypothesis of “more transmissible strains” selected through generational CT. In our study, we also observed a clear association between familiar history and CT. Women who confirmed infection in their mother had higher CT in their children, showing more frequency of persistence through generations when mediated by the congenital route of transmission. Moreover, the antecedent of infection in another sibling had a significantly strong association with the CT rate, implying an increase in risk from 8% to 36%. In socio-sanitary terms, this dynamics is noteworthy and requires that once a case is detected proactive interventions with a familiar approach are assumed, to assure diagnosis and early treatment of other cases of CD.

Several studies have shown the relevance of immune factors in the parasite-host equilibrium and in the modulation of parasitemia levels^6^ ^,^ ^17^ ^,^ ^28^ and the passage or infection of the placenta^13^ ^,^ ^36^ ^,^ ^37^, which largely determines the occurrence of CT. These immunological characteristics would be shared at the family level^6^ ^,^ ^38^ and contribute to the multifactorial network that determines the “cluster effect” proposed by Sanchez-Negrette et al.^28^, which we observed in epidemiological terms in this study.

Another noteworthy observation was the low CT rate in women from Santiago del Estero, a province with historically high levels of endemicity. There are no previous records of such difference in CT rates between the provinces of Argentina, even comparing with provinces as Chaco, which has a similar level of endemicity. This observation could lead to the hypothesis of differences in the circulating strains or other biological, environmental, and social characteristics of the population, which would contribute to attenuation in the virulence of the parasite^13^ ^,^ ^36^ ^,^ ^37^ or to a development of an equilibrium with the parasite in women, which would prevent the transmission during pregnancy. Another hypothesis is related to the administration of trypanocidal treatment through campaigns in this province during the 1990s (data not published, mentioned by Dr. Sosa-Estani), which may have benefited girls who are now mothers, who were still seroreactive but under the protective effect of the treatment^18^ ^-^ ^22^. As the antecedent of trypanocidal treatment was obtained based on the answers of the women included in the study, there could be a memory bias considering that trypanocidal was administered in these women during their early years.

In a broader view, a special dynamic of transmission would be operating in urban contexts and non-endemic areas, where infection is only mediated by CT and in which parasitic and immune factors would be shared and transmitted through generations, and may also be affected by environmental and social factors. All these factors continue under study and may allow us better comprehension of CT events and its manifestation at ecological level. 

This work contributes with observations in a large sample of contemporary population in an urban context of Argentina, which is the country with the highest absolute number of people with CD. These observations reinforce the hypotheses of parasitic and family characteristics leading to “cluster effect” and higher risk for CT, specifically observed in non-endemic and urban areas. Understanding CT in the new epidemiological scenario will allow us to reach a better approach to reduce its impact as a public health problem with global extent.
